# What matters to program partners when implementing a community-based exercise program for people post-stroke? A theory-based qualitative study and cost analysis

**DOI:** 10.3389/fresc.2023.1064206

**Published:** 2023-08-14

**Authors:** Gayatri Aravind, Kainat Bashir, Jill I. Cameron, Mark T. Bayley, Robert W. Teasell, Jo-Anne Howe, Alda Tee, Susan B. Jaglal, Susan Hunter, Nancy M. Salbach

**Affiliations:** ^1^ Department of Physical Therapy, University of Toronto, Toronto, Ontario, Canada; ^2^ Community Programs and After Stroke, March of Dimes Canada, Toronto, Ontario, Canada; ^3^ Department of Occupational Science and Occupational Therapy, University of Toronto, Toronto, Ontario, Canada; ^4^ The KITE Research Institute, University Health Network, Toronto, Ontario, Canada; ^5^ Toronto Rehabilitation Institute, University Health Network, Toronto, Ontario, Canada; ^6^ Schulich School of Medicine and Dentistry, Western University, St. Joseph’s Healthcare London—Parkwood Institute, London, Ontario, Canada; ^7^ Central East Stroke Network, Royal Victoria Regional Health Centre, Barrie, Ontario, Canada; ^8^ School of Physical Therapy, Western University, London, Ontario, Canada

**Keywords:** community exercise, cost, implementation, consolidated framework for implementation research, theoretical domains framework, balance, mobility, stroke

## Abstract

**Background:**

Community-based exercise programs integrating a healthcare-community partnership (CBEP-HCP) can facilitate lifelong exercise participation for people post-stroke. Understanding the process of implementation from multiple perspectives can inform strategies to promote program sustainability.

**Purpose:**

To explore stakeholders' experiences with undertaking first-time implementation of a group, task-oriented CBEP-HCP for people post-stroke and describe associated personnel and travel costs.

**Methods:**

We conducted a descriptive qualitative study within a pilot randomized controlled trial. In three cities, trained fitness instructors delivered a 12-week CBEP-HCP targeting balance and mobility limitations to people post-stroke at a recreation centre with support from a healthcare partner. Healthcare and recreation managers and personnel at each site participated in semi-structured interviews or focus groups by telephone post-intervention. Interviews and data analysis were guided by the Consolidated Framework of Implementation Research and Theoretical Domains Framework, for managers and program providers, respectively. We estimated personnel and travel costs associated with implementing the program.

**Results:**

Twenty individuals from three sites (4 recreation and 3 healthcare managers, 7 fitness instructors, 3 healthcare partners, and 3 volunteers) participated. We identified two themes related to the decision to partner and implement the program: (1) Program quality and packaging, and cost-benefit comparisons influenced managers' decisions to partner and implement the CBEP-HCP, and (2) Previous experiences and beliefs about program benefits influenced staff decisions to become instructors. We identified two additional themes related to experiences with training and program delivery: (1) Program staff with previous experience and training faced initial role-based challenges that resolved with program delivery, and (2) Organizational capacity to manage program resource requirements influenced managers' decisions to continue the program. Participants identified recommendations related to partnership formation, staff/volunteer selection, training, and delivery of program activities. Costs (in CAD) for first-time program implementation were: healthcare partner ($680); fitness coordinators and instructors ($3,153); and participant transportation (personal vehicle: $283; public transit: $110).

**Conclusion:**

During first-time implementation of a CBEP-HCP, healthcare and hospital managers focused on cost, resource requirements, and the added-value of the program, while instructors and healthcare partners focused on their preparedness for the role and their ability to manage individuals with balance and mobility limitations.

**Trial Registration:** ClinicalTrials.gov, NCT03122626. Registered April 17, 2017—Retrospectively registered, https://www.clinicaltrials.gov/ct2/show/NCT03122626

## 1. Introduction

Community-based exercise programs (CBEPs) adapted for people with stroke are emerging worldwide (1–7). Availability of these programs has the potential to facilitate a smooth transition from formal healthcare settings to the community *via* program referral. Continuing to exercise through CBEPs after rehabilition can help people post-stroke maintain their functional independence, feel better, and reduce the need for caregiver assistance (4, 7–9).

One novel program model is a community exercise program that incorporates a healthcare-community partnership (CBEP-HCP) (10). Programs based on this model have been evaluated in many countries, including Italy (11), the United Kingdom (12, 13), Canada (6, 7, 10), France (5), and the United States (2). In this model, healthcare professionals are involved with fostering referrals, and training and supporting fitness instructors to deliver an exercise program tailored to the needs of people post-stroke in a variety of community settings (14). The involvement of a healthcare professional appears to strengthen the credibility, safety, and quality of the exercise program (7, 15, 16).

Despite the documented benefits of exercise programs adapted for people post-stroke, these programs are not widely available in community settings (1, 17). This is partly due to the challenges with implementing CBEPs that ultimately lead to the discontinuation of some programs (1). Implementation science experts emphasize the need to understand the barriers and facilitators associated with the process of implementing programs to help plan approaches to optimize the success of initial implementation and, ultimately, program sustainability (18). It is particularly important to understand the implementation process from multiple stakeholders' perspectives given the complexity of exercise program models.

The use of theoretical frameworks to guide the investigations of implementation experiences is recommended as such frameworks provide a standard terminology and comprehensive overview of factors affecting the implementation process (19–21). The Theoretical Domains Framework (22) (TDF) and Consolidated Framework of Implementation Research (23) (CFIR) are frameworks that outline a set of multi-level determinants of implementation (23, 24). The TDF and CFIR have been effectively and extensively used in combination to understand implementation processes and address multiple study purposes (25). CFIR provides a comprehensive overview of organizational factors influencing implementation relevant to decisions made at the organization level to adopt a CBEP-HCP, whereas the TDF emphasizes individual-level factors relevant to the experiences of individual staff with program delivery.

Few studies have evaluated experiences of program providers with implementation of CBEPs post-stroke (1, 3, 4, 26–29). Organization-level challenges noted included resources to deliver the exercise class, program marketing, maintaining program integrity, sustaining partnerships, and funding (1, 3, 26, 28). Registrants with stroke identify transportation and program costs as barriers to participating in structured exercise programs (1, 4, 5, 7, 9, 30, 31). Recreation providers describe identifying fitness instructors that have the skill set to run these programs and staff turnover as implementation challenges (1, 27). Little attention has been paid, however, to experiences with first-time implementation of CBEPs that could help inform initial planning efforts. Also, despite the identification of program cost as a primary barrier to implementation, few studies (1) have explored the perspectives of healthcare and recreation centre managers who are responsible for addressing budgetary issues related to CBEP-HCP implementation, or incorporated use of theoretical frameworks to guide study design and interpretation of findings. Understanding the experiences of diverse stakeholders involved with the initial phases of implementation of CBEP-HCPs (i.e., decision to implement, partnership formation, delivery of first program) using a theory-based approach is critical to improving future plans to implement and sustain these programs.

Current guidelines (32) recommend a high level of supervision for group CBEPs involving people post-stroke. This means that delivering CBEPs tailored for people with stroke requires more human resources and a higher personnel cost than exercise programs designed for the general public. Equipment costs of CBEP implementation are not significant. This is because current guidelines (32) recommend exercise based on the practice of functional tasks within CBEPs for people post-stroke which requires minimal, low-cost equipment (e.g., chairs, aerobics steps) that are commonly available in recreation settings. Despite the important influence of cost on decisions to start-up a CBEP adapted for people post-stroke, few studies have attempted to estimate CBEP cost. One study estimated the cost of a CBEP involving 10 group exercise sessions with up to 2 trainers and 8 clients, and individual orientation and closing sessions (3). The cost per participant of providing the program ranged from £615 to £972. A breakdown of costs related to implementation was not provided (3). A detailed breakdown of personnel costs for recreation and healthcare organizations associated with the planning and first-time delivery of a single CBEP-HCP as well as travel costs for exercise participants, would potentially inform the decisions of recreation and healthcare managers to partner and implement the CBEP-HCP model.

We conducted a 2-group pilot randomized controlled trial to evaluate a CBEP-HCP called the Together in Movement and Exercise (TIME™) program at three urban sites in Ontario, Canada (6). TIME™ is a 12-week group, task-oriented exercise program targeting balance and mobility. At each site, a partnership between a hospital with designated stroke rehabilitation beds and a recreation centre was formed. The recreation centre provided the space, equipment, and fitness instructors to deliver the TIME™ program. The hospital provided a source of patients post-stroke for program referral and a healthcare professional to serve as the TIME™ healthcare partner who supported program delivery.

As part of the trial, we conducted a planned qualitative study to explore stakeholders' experiences with implementing the 12-week TIME™ program for people post-stroke for the first time. The implementation process included the decision to partner, training, coordination, and delivery of the first program. A secondary aim was to outline potential personnel and travel costs for program providers, healthcare providers, and exercise participants associated with first-time program implementation.

## 2. Materials and methods

### 2.1 Study design

A qualitative descriptive study involving semi-structured telephone interviews and focus groups was undertaken as part of pilot randomized controlled trial (6). The trial methodology is described in detail elsewhere (6). We used the consolidated criteria for reporting qualitative research (33) (COREQ) checklist to guide reporting. The research ethics board at the University of Toronto and at each participating hospital site approved the study protocol.

### 2.2 Program partners and recruitment

Table 1 describes stakeholder qualifications and responsibilities. Across the three study sites, 5 recreation managers, 3 healthcare managers, 8 fitness instructors, 3 healthcare partners, and 3 volunteers (1 site did not employ volunteers) were involved with TIME™ program implementation during the study and available for recruitment. On completion of the 12-week TIME™ program in the experimental group at a site, the central study coordinator contacted each stakeholder by email and invited them to participate.

**Table 1:** Stakeholder qualifications and responsibilities.

Stakeholder group	Setting	Qualifications and/or Responsibilities
Healthcare manager	Healthcare centre	Responsibilities: Management of budgets, staff, and healthcare services. Identifies the healthcare partner.
Recreation manager	Recreation centre	Responsibilities: Management of budgets, staff, and recreation services. Identifies fitness instructors to deliver the program.
Healthcare partner	Healthcare centre	Qualifications: Registered with their professional regulatory body with at least one year of experience treating people with stroke. Selected by healthcare managers. Responsibilities: Training volunteers. Completing 5 TIME™ class visits and providing feedback on class delivery to fitness instructors after the class; addressing instructor queries by email/phone; and promoting referral.
Fitness instructor	Recreation centre	Qualifications: CanFitPro™ Fitness Instructor Specialist, YMCA-Fitness Leadership, Ontario Fitness Council (OFC), American Council on Exercise (ACE), or equivalent; excellent communication and leadership skills; empathy, enthusiasm, and a genuine interest in working with people with mobility issues Responsibilities: Training and supervising volunteers (if applicable); teaching the exercise program.
Volunteer	Recreation centre	Qualification: Experience with exercise; knowledge of anatomy, and nutrition, or related fields; and an interest in working with individuals with mobility issues. Responsibilities: Under the supervision of fitness instructors, setting up and removing equipment; running the walking station.

### 2.3 Together in movement and exercise (TIME™) program

The TIME™ program offers two one-hour exercise classes per week for 12 weeks. Group classes include a seated warm-up, repetitive and progressive practice of functionally relevant balance and mobility tasks organized in a 3-station circuit, and a seated cool-down. An instructor-to-participant ratio of 1-to-4 is required to optimize supervision and safety; volunteers can be used to supplement instructors to attain this ratio. Healthcare partners visited the first two classes and three additional classes interspersed across the remaining 22 classes (6).

In the trial, community centres first signed the TIME™ license, and then received an electronic toolkit with materials (e.g., liability waiver, exercise guidelines, equipment list, etc.) required to implement the program. Fitness instructors, healthcare partners, and volunteers completed training which involved a combination of e-modules, and in-person and virtual sessions (6).

### 2.4 Data collection

We conducted interviews with managers, and focus groups, consisting of two to three participants, with fitness instructors or volunteers. A Toronto-based, female, interviewer with six years of experience with conducting qualitative research obtained informed consent and conducted focus groups and interviews. Interview or focus group guides were tailored to each stakeholder group. We selected the CFIR to inform interview guides for managers as this framework incorporates factors at the organizational level relevant to managers’ experiences and decision-making. We selected the TDF to inform the interview and focus group guides tailored for program providers as this framework emphasizes factors relevant to individual experiences and decision-making. Across sites, interviews and focus groups were held up to 8 months post-intervention and lasted 45–60 min. The interviewer used probing questions to obtain greater detail or explanation. Sessions were conducted by telephone to allow for flexibility given geographical distance. Sessions were audio recorded and professionally transcribed.

To inform the description of costs, we documented the time required to complete the activities involved with training and delivery of the first program. We also documented the hourly wages of the staff involved in program delivery and the cost of the equipment that was purchased for the sites. We requested fitness instructors to document the travel modes used by participants to attend the class using a standardized form, while healthcare partners were requested to document the time spent on class visits.

### 2.5 Qualitative analysis

A directed content analysis that incorporated the CFIR and TDF was undertaken. We developed a codebook composed of CFIR and TDF domains. Two researchers (NMS and KB) used an inductive and deductive approach to independently open-code one transcript per stakeholder group (five in total). Researchers then met to compare and contrast codes to ensure consistency and to revise the coding scheme before KB proceeded to independently code the remaining transcripts using NVivo 10 software. Subsequently, for each CFIR and TDF domain, KB reviewed coded text from all stakeholder groups first within each site and then compared and contrasted coded text across sites. NMS, GA, and KB met and reviewed within-site node summaries to identify emerging categories as they related to facilitators, barriers, and recommendations for the process of first-time implementation of the exercise program. After recognizing that experiences were similar across sites, KB combined site-specific data to develop summaries of facilitators and barriers within each CFIR and TDF domain, and recommendations, with supporting quotes. Through a series of meetings, NMS, GA, and KB met and refined summaries of the facilitators and barriers to ensure alignment with CFIR and TDF domains. An inductive approach was then used to identify overarching themes across the data through discussion and consensus. Similarly, the recommendations and supporting quotes were reviewed and classified by the activity within the implementation process. To maintain records and encourage reflexivity about data collection procedures, an audit trail was used. The interviewer took reflexive notes after completing each session related to similarities across interviews and focus groups and new ideas arising from the data. Preliminary results were presented and discussed with the interdisciplinary research team to consider alternative interpretations of the data. Throughout the analysis, discrepancies around emerging ideas, codes, and themes were resolved by discussion and through reference to the original transcripts to ensure authors were in agreement. Lastly, we presented findings with direct quotations from the participants as exemplary anecdotes that support the findings of the study.

### 2.6 Cost analysis

We developed a sample budget that presents personnel and travel costs for the healthcare partner, recreation partner and the participant to run a 12-week TIME™ program. For healthcare and recreation partners, we distinguished costs for planning and implementation activities. We took the mid-point value of pay rates observed across the three sites during the study to generate cost estimates; otherwise we used and indicated an alternate source.

## 3. Results

Twenty individuals from three study sites participated in focus groups or interviews. Participants included 4 recreation managers, 3 healthcare managers, 7 fitness instructors, 3 healthcare partners and 3 volunteers. Fitness instructors had between 8 and 30 years of experience in their position. Healthcare partners were physical therapists with between 16 and 30 years of clinical experience. Each healthcare partner completed five planned class visits.

In site A, a municipal for-profit recreation centre partnered with a local hospital. In site B, a hotel-based for-profit fitness centre partnered with a local hospital and in site C, a non-profit recreation centre partnered with a local hospital.

The experiences of managers and program staff could be grouped into the following two categories based on the phase of program implementation: (1) experiences with the decision to partner and implement the program; and (2) experiences with training and program delivery.

Within each category two main themes emerged. Table 2 provides a summary of the results and the associated CFIR and TDF domains under each theme.

**Table 2:** Experiences of managers and program staff with program implementation.

Theme	CFIR domain(s)	TDF domain(s)	Theme description
*Experiences with the decision to partner and implement the program*			
Program quality and packaging, and cost-benefit comparisons influenced managers’ decisions to partner and implement the CBEP-HCP • Program quality and packaging • Comparison of program cost to potential benefits	Intervention characteristics - Intervention source - Evidence strength and quality - Relative advantage - Design quality and packaging - Cost Process - Engaging: external change agents Inner setting - Readiness for implementation: available resources - Implementation climate: compatibility		Managers identified key factors that facilitated decision to implement program: • Reputation of program developers • Scientific basis of program • Successful implementation of program in other centres • Involvement of healthcare professionals to support instructors • Program introduction by knowledgeable individuals • Program introduced to audience that includes decision makers and representatives of program staff Managers recognized apparent benefits to program implementation: • Opportunities for staff to acquire specialized knowledge and training from healthcare professional (recreation manager) • Opportunity for organization to partner with local healthcare organization (recreation manager) • Opportunity to offer services to and positively impact seniors and individuals with disability living in the community (recreation manager) • Ability to schedule program during facility's “slow time” (recreation manager) • Ability to offer patients alternative means to engage in exercise after exiting hospital and rehabilitation services (hospital manager) • Opportunity to be involved with community programs and be perceived as leaders in stroke care (hospital managers) • Provision of funding to cover costs related to training, program delivery, and equipment (recreation and hospital manager)
Previous experiences and beliefs about program benefits influenced staff decisions to become instructors		Knowledge - Procedural knowledge Skills - Skills Beliefs about consequences - Outcome expectancies	Decision to participate as instructors was influenced by: • Previous experience working as instructors for people with cognitive or motor impairments • Opportunity to gain experience working with clients who have mobility issues, in a group setting • Opportunity to advocate for the benefits of exercise to a new group of individuals Decision to participate as a healthcare partner was influenced by: • Opportunity to learn about a novel program • Opportunity to educate patients and refer them to the program • Opportunity to influence how the program is run
*Experiences with training and program delivery*			
Program staff with previous experience and training faced initial role-based challenges that resolved with program delivery		Knowledge - Procedural knowledge Skills - Skills Beliefs about capabilities - Self-efficacy Emotion - Anxiety Environmental context and resources - Resources/Material resources Social/Professional role and identity - Professional role Beliefs about consequences - Outcome expectancies	• TIME™ program training, toolkit, and online support videos described as comprehensive and useful • Fitness instructors faced challenges in understanding stroke-related deficits and grouping participants based on ability levels • Instructors felt nervous about managing individuals with mobility issues especially as non-healthcare professionals • Instructors desired information about participants’ mobility aid use and falls risk • Challenges included the availability of equipment and a lack of volunteers support with participants with low ability levels • Instructors and healthcare partners gained confidence over time • Healthcare partners and fitness instructors requested additional clarity in roles, and emphasized the need for teamwork to empower participants.
Organizational capacity to manage program resource requirements influenced managers’ decisions to continue the program	Inner setting - Readiness for implementation: available resources		During program delivery: • Managers monitored and were concerned with ensuring availability of adequate financial, human, and space resources • Managers could not identify fitness instructors with previous experience of working with people with disability in a group setting

### 3.1. Experiences with the decision to partner and implement the program

#### 3.1.1. Theme 1: Program quality and packaging, and cost-benefit comparisons influenced managers' decisions to partner and implement the CBEP-HCP

##### 3.1.1.1. Program quality and packaging

When making a decision to partner with a novel program, healthcare and recreation managers strongly considered the quality of the program as reflected by the reputation of the organization that developed it, presence of scientific evidence, and evidence of its successful implementation. These factors influenced managers' opinions of the value of implementing the program.

Recreation managers also confirmed the positive impact of the method of program introduction. Involving a knowledgeable individual to introduce and highlight the advantages of the program was critical to ensure interest and support, as was the presence of all organizational decision-makers and managers during the meeting.

*[The lead investigator] did all of her homework … approached the program director and then was invited for an information session. And during the information session, she made sure that all the right players were at the table—the manager who would be involved, and the program directors … So when she did the presentation and provided all of the information … Because she was very thorough, it was a no-brainer that this is something that as an organization we should be supporting. (Site A, ID1, Hospital manager)*

##### 3.1.1.2. Comparison of program cost to potential benefits

Managers reported that the potential benefits of the program for their clients and their organization were important in their decision to adopt the program. Recreation managers recognized that their staff involved in the program would acquire new skills and knowledge to support people with mobility issues, and that the model offered novel opportunities to partner with reputable research and healthcare organizations. They also valued the opportunity to positively impact the lives of marginalized seniors residing in their communities in addition to establishing their presence in the rehabilitation sector.

Hospital managers viewed their involvement with a novel community program as supporting their organizational mandates to be leaders in stroke care. They viewed the TIME™ program as serving their outgoing patients by enabling them to continue advancing their recovery in the community setting. Recreation and hospital managers perceived the compatibility of the program and organizational values and goals as another facilitator of their decision to adopt the program.

Managers also identified cost and resource requirements of the program as critical factors to consider. Hospital managers weighed the amount of time needed from the physiotherapist for initial training and program visits. They were concerned about the ability of the physiotherapist to manage their role as a healthcare partner along with their clinical responsibilities. Recreation managers viewed the recommendation to schedule the program during non-peak hours as advantageous as staff and resources could be made available. Recreation and hospital managers acknowledged that the provision of funding to support the ongoing involvement and training of their staff was crucial to their wilingness to partner with and implement the CBEP-HCP.

*So what is this going to cost? There aren’t any extra dollars available within the healthcare system and within our organization. So had there been a major cost to this then we would have to weigh the benefit to the patient and the cost associated to that to see whether or not it could be made a priority for us. (Site B, ID4, Hospital manager)*

#### 3.1.2. Theme 2: Previous experiences and beliefs about program benefits influenced staff decisions to become instructors

Program delivery staff described feeling excited about taking part in the TIME™ program and viewed their participation as an opportunity to gain experience working with clients who have mobility issues, and advocate for the benefits of exercise. They associated their interest in becoming TIME™ program instructors with their previous experience prescribing exercise for and working with people with stroke or cognitive or motor impairment. Healthcare partners saw their involvement as an opportunity to learn about a novel program so they could not only educate their patients and refer them to the program, but also influence how the program is run.

*I think it's just great to see the potential for a community program for the stroke patients. So that we're aware of what's being offered out there… I love being with the patients. And it is an opportunity to educate them—the fitness instructors as well. I try to give a little bit of information where I can. I mean it benefits everybody. (Site B, ID20, Healthcare partner)*

### 3.2. Experiences with training and program delivery

#### 3.2.1. Theme 1: Program staff with previous experience and training faced initial role-based challenges that resolved with program delivery

Fitness instructors, who had experience working with people with mobility issues, and healthcare partners, found the TIME™ program training, toolkit and online support videos comprehensive and useful. However, as first-time instructors for the TIME™ program, they reported initial challenges with understanding the impact of stroke-related deficits and grouping participants based on their ability levels. They also described feelings of nervousness in managing individuals with mobility issues in a group format and raised concerns about their abilities to manage larger groups with varying ability levels in the future. Some of this uncertaintly was attributed to the lack of confidence in managing individuals who may need additional support while not being a healthcare professional. As one instructor explained:

*So I was a little nervous about it. Like having these people sort of under my wing, like being responsible for… Like when you're not a physiotherapist or you're not a doctor or a nurse or a healthcare provider but just as an instructor, like a yoga instructor, I just felt like I was a little bit out of my element maybe. Only because of the responsibility part. I feel like it was a pretty big responsibility to be able to guide these people. (Site C, FG, ID14, Fitness instructor)*

Other instructors commented that additional training and material may be required for fitness instructors with minimal exposure to individuals with mobility issues. Although centres were provided with information on mobility aids and medications used by participants in advance of the program, instructors expressed a desire for information about mobility aid use and falls risk before the first class so they could understand the diverse ability levels of participants.

Some instructors reported difficulty in recalling all the exercises and levels within the circuits and used cue cards as aids. Other challenges related to the availability of resources. At one site, instructors reported not having access to optimal ballet bars or chairs for handholds, or not having enough volunteers to assist participants with low ability levels.

Intructors noted, however, that they gained confidence and grew more comfortable over time with the experience of delivering more classes; a change also reported by healthcare partners. The healthcare partners confirmed observing these changes in the instructors and ascribed it to the instructors gaining an understanding how stroke deficits may affect a person' ability to do different exercises, and getting more comfortable with the circuits and running a group class.

*And so I think the first couple of sessions when they start working with stroke patients, that’s when they start to click and think, “Oh, okay, so that’s what they mean.” It’s just they don't have the background, right. (Site A, ID7, Healthcare partner)*

Fitness instructors and healthcare partners reflected on their professional roles and highlighted the importance of being able to work with people, troubleshoot and empower each other. They noted the need to work as a team where the healthcare partners, instructors and volunteers support each other to be able to work with participants with different abilities and challenges; which in turn would allow them to support and empower their participants.

*[Healthcare partners have] to be comfortable dealing with people, and feel comfortable troubleshooting. You definitely can't have an ego and say that yeah, I'm the physio … here it's all about empowerment—empowering the patient, empowering the instructors to make sure they can be a partner in helping the patient help themselves, and empower the volunteers so they can feel a part of this program. (Site A, ID7, Healthcare partner)*

Program providers also expressed the need for improved clarity in their roles. Healthcare partners discussed the need to recaliberate their professional role and identity from a hands-on, clinical role to more of an observer or supervisory role. At one site, the healthcare partner noted they felt instructors viewed them as a supervisor during class visits, and the healthcare partner was not comfortable in this role. One healthcare partner reported that inadequate guidance was provided on the responsibilities of the instructors and the healthcare partner to group registrants for the exercise stations during the first class. Fitness instructors reported the need to guide the volunteers on what to do during the class, and questioned if this was a part of their role as an instructor and pointed to the need for more information during volunteer training.

#### 3.2.2. Theme 2: Organizational capacity to manage program resource requirements influenced managers' decisions to continue the program

All managers identified availability of adequate financial, human, and space resources as factors to consider for program maintenance and continuity. Recreation managers emphasized the need to have more than two instructors available to ensure that classes would not be cancelled due to illness. Although fitness instructors with experience working with people with mobility issues in a group setting were desired, some fitness instructors only had experience working with individuals with mobility issues one-on-one.

*One of the factors was availability of instructors. So you never want to run a program just with one or two. Just that if there's any sickness or anything comes up … our goal is never to cancel a class … And then space is obviously a major consideration. (Site C, ID19, Recreation manager)*

Hospital managers reported concerns related to the time and the cost commitment required from the hospital for the healthcare partner to perform their duties (training fitness instructors and volunteers, consultation and supervision). The managers highlighted the potential impact of redirecting manpower from the hospital and its impact on the professional commitments at the hospital. One hospital manager reflected on the need to identify a suitable individual for the role.

*We had to take into account how much time was going to be needed from the physiotherapist at our organization …And so the training up front, the cost of the training, the cost for her time was a big piece. And then even aside from the cost, it was do we even have the manpower?… It's often difficult to recruit physiotherapists. So we wanted to make sure that we actually had the person that could give up time to do this. (Site B, ID4, Hospital manager)*

### 3.3 Recommendations

Table 3 presents recommendations made by participants on how to improve the process of implementating the TIME™ program. Recommendations related to engaging healthcare and recreation organizations, selecting staff and volunteers, training, healthcare partner visits and referral, intake, and class instruction.

**Table 3:** Recommendations for TIME™ program implementation.

Target stakeholder	Activity	Recommended approach
TIME™ implementation team	Engaging healthcare and recreation organizations	• If possible, identify healthcare and recreation organizations with a pre-existing relationship to facilitate the decision to partner • A TIME™ expert should make initial contact by email and attach a brief overview of the program • Subsequently schedule a face-to-face introductory meeting to build rapport; provide supporting documents in advance • Include healthcare/recreation personnel with decision-making power during introductory meetings • A representative who is knowledgeable about the TIME™ program should attend the introductory meeting • To persuade the partner, emphasize: 1) how the TIME™ program aligns with the organization's mandate, strategic plan, or priorities; 2) research evidence (eg journal article) supporting program safety, feasibility, and benefits; 3) number of centres successfully running the program; 4) evidence of credibility and commitment of potential partnering organizations; 5) how the TIME™ program meets patient needs/benefits clients; 6) the resources required and a budget giving a general idea of the potential costs.
	Engaging the healthcare organization	• To persuade the partner, emphasize: 1) how the TIME™ program provides an exit strategy that may facilitate discharge.
	Engaging the recreation organization	• To persuade the partner, emphasize: 1) the role of the healthcare partner to support fitness instructors; 2) the opportunity for fitness instructors to build new skills; 3) the opportunity to reach a marginalized clientele.
	TIME™ training workshop	• Clearly define the roles of people involved with delivering the program (i.e., healthcare partners, fitness instructors and volunteers) • Incorporate team activities during the program training to build rapport • Clarify how fitness instructors and the healthcare partner will interact during the first class • Provide regular training opportunities to address staff turnover
Healthcare manager	Selecting healthcare partners	• Select a healthcare partner who is adaptable and enjoys educating and empowering others
	Training healthcare partners	• Consider training 2 healthcare partners per program to optimize availability and minimize impact of class visits on patient care • The healthcare partner should attend the TIME™ training with fitness instructors to learn about roles, become familiar with the exercises, and establish rapport with the fitness instructors
Healthcare partner	Visiting the first two classes	• Fitness instructors and the healthcare partner should meet before the program starts to confirm what each person will do during the first class visit • The healthcare partner should bring the exercise guideline for reference purposes for class visits
Recreation manager or Fitness coordinator	Selecting fitness instructors	• Select fitness instructors with experience instructing people with mobility issues to exercise in a group format
	Training: content, timing, and number of staff to train	• Recreation providers should ensure that 4 fitness instructors complete TIME™ training for an expected class size of 8 in case of staff unavailability and/or turnover • Training should ideally be completed within a few weeks of the program start date • If possible, fitness instructors should shadow an ongoing TIME™ program or meet patients post-stroke to familiarize them with potential clients
	Intake	• Fitness coordinators should screen participants to ensure the program is appropriate for their ability level • Fitness coordinators should ensure that fitness instructors are familiar with adverse events protocols
	Prior to first class	• Fitness coordinators should provide fitness instructors with information on participant mobility level (e.g. mobility aid used) prior to the first class to help them prepare to group individuals
	Selecting volunteers	• Select volunteers with a positive attitude towards helping others
Fitness instructor	Teaching the TIME™ program for the first time	• Class size should be smaller than the maximum of 12 to give fitness instructors time to become familiar with the program with a lower supervision burden • Fitness instructors may find cue cards that describe the exercises at each super station helpful in the first few classes • Fitness instructors should be particularly cautious supervising new participants by remaining close by and asking participants to move slowly
	Adjusting the difficulty level of exercises	• Fitness instructors should increase or decrease the difficulty level of the exercises to match the ability level of the participant to ensure safety; the ability to achieve a higher difficulty level can contribute to a sense of accomplishment
Volunteer	Volunteer training	• Volunteers should view videos of TIME™ participants before the scheduled training session

### 3.4 Estimated personnel and travel costs to run a 12-week TIME™ program

Table S1 in Supplementary file S1 presents a sample budget of estimated personnel and travel costs for the planning and first-time implementation of a 12-week TIME™ program (two classes per week) for a group of 12 participants with two fitness instructors and one volunteer per class. For the healthcare centre, the estimated total cost of the healthcare partner, including selection, training, class visits, and consultations, is $680 CAD. For the recreation centre, the estimated total cost of running one program, including purchasing the TIME™ license, and selection, training, and use of fitness instructors, is $3,153 CAD. Therefore, the sum total of healthcare and recreation costs was $3,833 CAD. The transportation cost for participants to attend the entire program is $283 CAD based on mileage for using a personal vehicle, and $110 CAD for taking public transit.

## 4. Discussion

Findings highlighted important considerations made by stakeholders during the decision to partner and implement the TIME™ program and during training and program delivery. Program quality and packaging strongly influenced the perceived value of the program among managers who also weighed program costs and required resources with expected benefits for staff, clients, and and their organization. Positive experiences during training and program delivery were facilitated by engaging fitness instructors who had experience working with people with disability and the comprehensiveness of the program training procedures and materials. Nevertheless, program staff faced personal challenges with taking on a new role that diminished as they gained experience delivering the program. Organizational capacity to manage program resource requirements influenced managers' decisions to continue the program. Findings yielded cost estimates and recommendations from multiple stakeholders that will help to optimize the process of first-time implementation.

### 4.1 Organizational perspectives on implementation

Recreation and healthcare managers reported similar priorities and organizational considerations that influenced their decision to implement the program. The reputation of program developers, scientific evidence, successful implementation in other centres, interaction with program experts, and alignment with organizational mandates, were influential. This suggests that even when implementing an evidence-based program such as the TIME™ program, management places importance on how it is implemented, who is involved with the implementation, and program compatibility with the characteristics of the organization and the community. Determining who presents the program is important as someone with comprehensive knowledge of the program can highlight key advantages of the program and address any concerns. When organizational leaders have confidence in the program and display a readiness for change, it is reflected in the program staff's attitudes and involvement (34) which in turn influences the participants' experiences with the program (35).

Recreation and hospital managers confirmed that organizational capacity to bear program costs, a commonly reported barrier to program implementation (17, 29, 36, 37), impacted their decision to deliver the program. Findings from this study add new information on the timing and extent of costs. When implementing a licensed CBEP-HCP, an established community recreation centre will encounter costs related to the license and instructor training and program delivery. If the recreation centre employs the healthcare partner, then additional costs would include healthcare partner training and visits. The healthcare partner costs steadily decline over time as instructors gain experience and skill, requiring fewer sessions of supervision/consultation (1). Training the instructors is a single time cost, but additional instructor training costs may arise if refreshers are needed or if new instructors require training because of program scale-up or staff turnover (1). The TIME™ program utilizes equipment that is commonly available in most recreation centres (38).

Community organizations can establish cost-recovery mechanisms to offset the cost of implementing and delivering the program. This includes program fees, membership fees, donations or applying for and obtaining funding to support program activities (1, 15, 29). Program duration may vary across sites, and can be further influenced by parameters such as use of volunteers or regional cost of living (1). Using the cost analyses presented in this study, community organizations can estimate the anticipated costs at the time of implementation based on their program design. For healthcare systems, supporting CBEP-HCPs through referrals and providing a healthcare partner has the potential for cost-savings in the long-term. The availability of local CBEP-HCPs may help to expedite discharge from outpatient services and decrease costs by reducing the duration of outpatient care. Participation of people post-stroke in community exercise programs may be associated with significant cost savings to the healthcare system resulting from the reduced use of healthcare services (39–41). Hospital managers and healthcare partners can consider these factors to justify the allocation of time and personnel resources towards such programs. The public transportation cost for participants is sufficiently high that it may pose a barrier to attending the program. Recreation centres should consider transportation cost and ability to pay when offering community exercise programs for people with stroke and provide subsidies or identify low-cost alternative transportation options in their local communities.

In this study, healthcare and recreation managers placed value on inner setting variables such as organizational readiness to implement the program, and the potential growth in organizational impact on the community brought on by program implementation. Since the program could be scheduled during “off-peak times”, it was not challenging for the recreation manager to allocate space, equipment, and instructors to the program. Interestingly, despite their mandate to include accessible programming for all community residents, it was the implementation of the TIME™ program that allowed the recreation organization to serve residents who experience balance and mobility limitations resulting from a stroke. This intersects with hospital managers' identified need for programs in the community that support people with stroke as they transition from the hospital to home. The partnerships created through the process of implementation were nurtured through the complementary mandates and a shared culture that values innovation and inclusivity. While some of these factors have been identified in previous studies on the implementation of CBEP-HCPs (1, 16), this is one of the first studies to present managerial perspectives. Understanding managers’ perspectives is critical because, in addition to overseeing allocation of staff and resources, managers are key decision-makers about program implementation, delivery and long-term sustainability (29).

### 4.2 Program provider perspectives on implementation

Program staff were aware of the influence of their skills, knowledge, and beliefs on the delivery and impact of the program. In the absence of previous experience working with individuals with disabilities, program staff described a lack of confidence in managing people of varying abilities. At the same time, their beliefs about the positive consequences of participating in the program for themselves (obtaining/expanding skills) and the participants (affecting a positive change in their health) motivated them to remain involved. Recommendations to improve support included adapting the training material to cater to inexperienced instructors and discussing specific challenges, such as lack of resources, participant safety, and delivering a class for people with varied ability levels. Importantly, program providers recognized having previous experience working with individuals with disabilities and the eagerness to work with them as important attributes in a TIME™ fitness instructor.

Study findings reinforce the role of the manager in identifying staff who fit the profile of a suitable instructor. This would include someone with experience and eagerness in working with individuals with mobility issues, ability to troubleshoot and innovate on-the-go, or the willingness to learn these skills. To assist instructors with program delivery, the healthcare partner role would require an individual who is able to empower and work with the instructor to adapt the program to the needs and abilities of the participants. When program staff can internalize the benefits of associating with the program and feel supported through the challenges, turnover may decline (42). This is important because participants rely on consistency and trust developed over time for their continued participation in community-based programs (43).

The factors identified as facilitators and barriers to first-time program implementation in the current study also impact program continuity and sustainability. In a study examining factors influencing sustainability of CBEP-HCPs (29), CBEP-HCPs that discontinued had common features including inexperienced instructors. Sites that sustained the CBEP-HCP for four or more years had common features including stable funding models, experienced and motivated instructors, supportive management, dedicated resources, support of a local champion and a strong network of local partners (29). Planning for these determinants at the time of implementation can set the stage for program sustainability.

## 5. Conclusion

During first-time implementation of a CBEP-HCP, healthcare and hospital managers focused on cost, resource requirements, and the added-value of the program, while instructors and healthcare partners focused on their preparedness for the role and their ability to manage individuals with balance and mobility limitations. Stakeholders identified recommendations that can be used to improve first-time implementation with people post-stroke. Personnel and transportation cost estimates can help inform decisions to implement the CBEP-HCP model with people post-stroke and highlight the need to identify low-cost transportation options for exercise participants in financial need.

## Data Availability

The datasets presented in this article are not readily available because participants did not consent to sharing their data.
