# Supplementary material for: What matters to program partners when implementing a community-based exercise program for people post-stroke? A theory-based qualitative study and cost analysis
Source: Front Rehabil Sci. 2023 Aug 14;4:1064206. doi: 10.3389/fresc.2023.1064206 (PMC10461472; doi:10.3389/fresc.2023.1064206)
Supplement: Supplementary file 1 [file Table1.pdf]

Supplementary File 1

Table 1. Sample budget for personnel and travel costs to run a 12-week TIME™ program for the first time

Program activity or component	Details	Position for budget item (if applicable)	Hourly pay rate (if applicable)	Source of pay rate	Hours	Cost (CAD)
**HEALTHCARE PARTNER**						
Planning phase						
Administrative preparation	Selecting healthcare partner $104,000 annual salary, 40-hour work week	Hospital manager	$50.00	Website [1]	2	$100.00
Training of 1 healthcare partner	$70,000 annual salary, 40-hour work week	Healthcare partner	$33.65	Published PT starting salaries [2]		
• Review exercise guideline	3 hours				3	$100.95
• Complete e-module	2 hours				2	$67.30
• In-person workshop	3.5 hours				3.5	$117.78
Implementation phase						
Class visits	2.25 hours/visit (1 hour travel, 1 hour class, 15-minute debrief), 3 visits/program*	Healthcare partner	$33.65	Published PT starting salaries [2]	6.75	$227.14
Consultation	Emails, phone calls (note: additional time required to promote referral)				2	$67.30
**SUB TOTAL**					**19.25**	**$680.47**
**RECREATION PARTNER**						
Planning phase						

Administrative preparation	Coordinating instructors, equipment, and rooms	Fitness coordinator	$30.00	Observed in study	4	$120.00
TIME^{TM} program license (electronic version)†	Includes TIME™ toolkit and trademark license		$100.00	Website [3]		$100.00
Review the TIME^{TM} toolkit		Fitness coordinator	$30.00	Observed in study	4.5	$135.00
Training	Train 4 fitness instructors to mitigate scheduling issues	Fitness instructor	$25.00	Observed in study		
• Complete e-module	2 hours/instructor x 4				8	$200.00
• In-person workshop	3.5 hours/instructor x 4				14	$350.00
• Practice as a group before first class (recommendation)	2 hours/instructor x 4				8	$200.00
Implementation phase						
Intake	15 min per person to screen 15 individuals by phone to fill a 12-person class	Fitness coordinator	$30.00	Observed in study	3.75	$112.50
Class delivery	2 instructors, 1.5 hours per class, 24 classes + 30 min per healthcare partner debrief x 3 debriefs	Fitness instructor	$25.00	Observed in study	75	$1,875.00
Administrative tasks	Managing participants, consultations with healthcare partner	Fitness coordinator	$30.00	Observed in study	2	$60.00
**SUB TOTAL**					**119.25**	**$3,152.50**
**PARTICIPANT TRANSPORTATION, COST PER INDIVIDUAL**						

Personal vehicle	10km per way,[‡] 2-way trip per class, 24 classes, mileage $0.59/km		$11.80 per class	Canada Revenue Agency [4]	24	$283.20
Public transit	Seniors (65+ years) fare: $2.30 per way, 2-way trip per class, 24 classes		$4.60 per class	Observed in study [5]	24	$110.40

[*]3 vists per program are currently recommended although 5 visits were made during the study.
[†]Hard copy $85.00 + shipping/handling (cost will vary based on location).
[‡]Common distance travelled [6]; parking was free at study sites.

References

1. Hospital manager average salary in Canada. (2022). https://ca.talent.com/salary?job=hospital+manager (Accessed October 6, 2022).
2. Ontario Physiotherapy Association New Grad Toolkit. (2021). https://opa.on.ca/membership/new-grad-toolkit-2021/ (Accessed October 6, 2022).
3. Together in Movement and Exercise (TIME^{TM}). https://www.uhn.ca/TorontoRehab/Clinics/TIME (Accessed October 6, 2022).
4. Government of Canada automobile allowance 2022. https://www.canada.ca/en/revenue-agency/services/tax/businesses/topics/payroll/benefits-allowances/automobile/automobile-motor-vehicle-allowances/automobile-allowance-rates.html (Accessed October 6, 2022).
5. Toronto Transit Commission fares and passes. https://www.ttc.ca/Fares-and-passes (Accessed October 6, 2022).
6. Alsbury-Nealy K, Colquhoun H, Jaglal SB, Munce S, Salbach NM. Referrals from healthcare professionals to community-based exercise programs targeting people with balance and mobility limitations: an interviewer-administered survey. *Physiother Can*. (2023). doi:10.3138/ptc-2022-0069.
